# Resolution of Cutaneous Old World and New World Leishmaniasis after Oral Miltefosine Treatment

**DOI:** 10.4269/ajtmh.2010.09-0490

**Published:** 2010-01

**Authors:** Dennis Tappe, Andreas Müller, August Stich

**Affiliations:** Institute of Hygiene and Microbiology, University of Würzburg, Würzburg, Germany; Department of Tropical Medicine, Medical Mission Hospital, Würzburg, Germany

**Figure 1.**
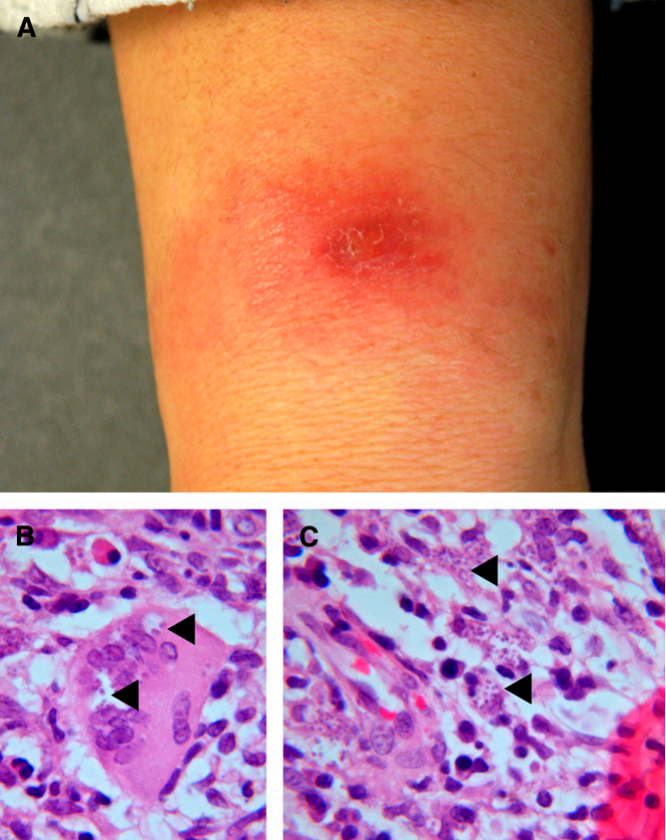
Cutaneous Old World leishmaniasis caused by *Leishmania tropica* in an Afghan woman. **A**, Initial presentation of the dry, plaque-like lesion with central nodule on the forearm. **B**, Intracellular parasites in vacuoles of a giant cell (arrowheads). Haematoxylin and eosin stain, magnification ×1,000. **C**, *Leishmania* in macrophages (arrowheads). Haematoxylin and eosin stain, magnification ×1,000. This figure appears in color at www.ajtmh.org.

**Figure 2.**
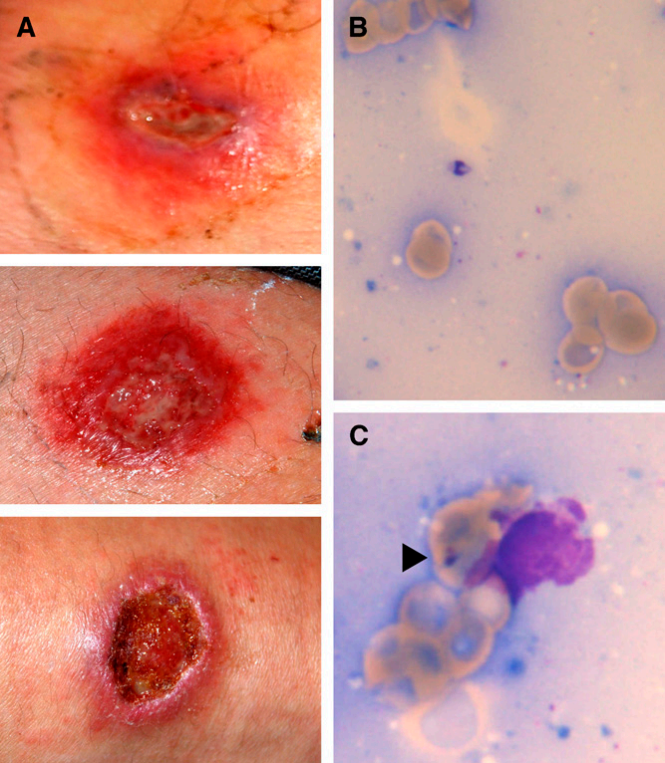
Cutaneous New World leishmaniasis caused by *Leishmania braziliensis* in a German traveler after a vacation in Costa Rica and Belize. **A**, Initial presentation of the wet ulcerated lesion on the ankle (upper row), thigh (middle row), and forearm (lower row). Lesions were superinfected with Panton Valentine leukocidin-negative *Staphylococcus aureus*, *Escherichia coli*, and viridans streptococci. **B**, Extracellular *Leishmania* amastigote in tissue scarification. Giemsa stain, magnification ×1,000. **C**, Intracellular parasite in a monocyte (arrowhead). Giemsa stain, magnification ×1,000. This figure appears in color at www.ajtmh.org.

**Figure 3.**
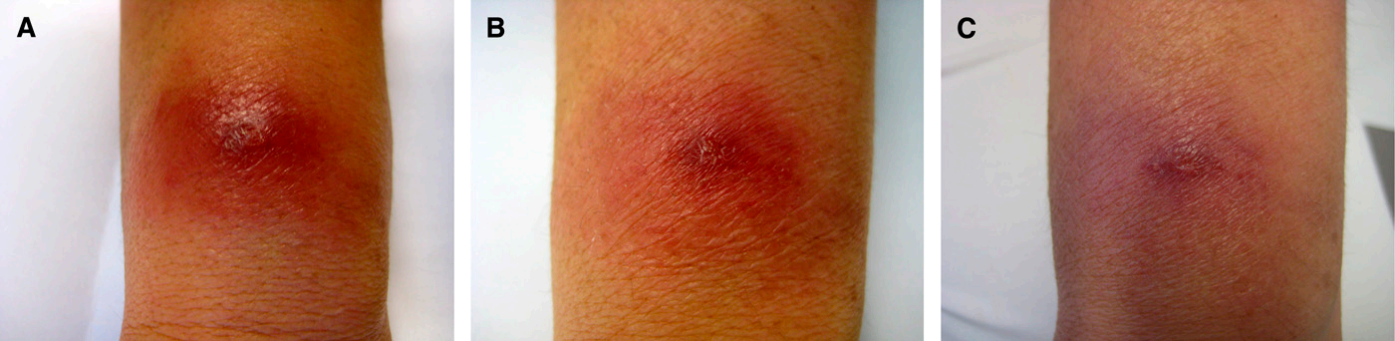
Follow-up presentation of Old World cutaneous leishmaniasis. **A**, Increasing local inflammation 14 days after start of miltefosine treatment. The lesion looks more nodular than before treatment. **B**, Decreasing inflammation 21 days after start of pharmacotherapy. **C**, Resolution of lesion 4 weeks after the end of treatment. Repeated *Leishmania* serology remained negative throughout the observation period in this patient. This figure appears in color at www.ajtmh.org.

**Figure 4.**
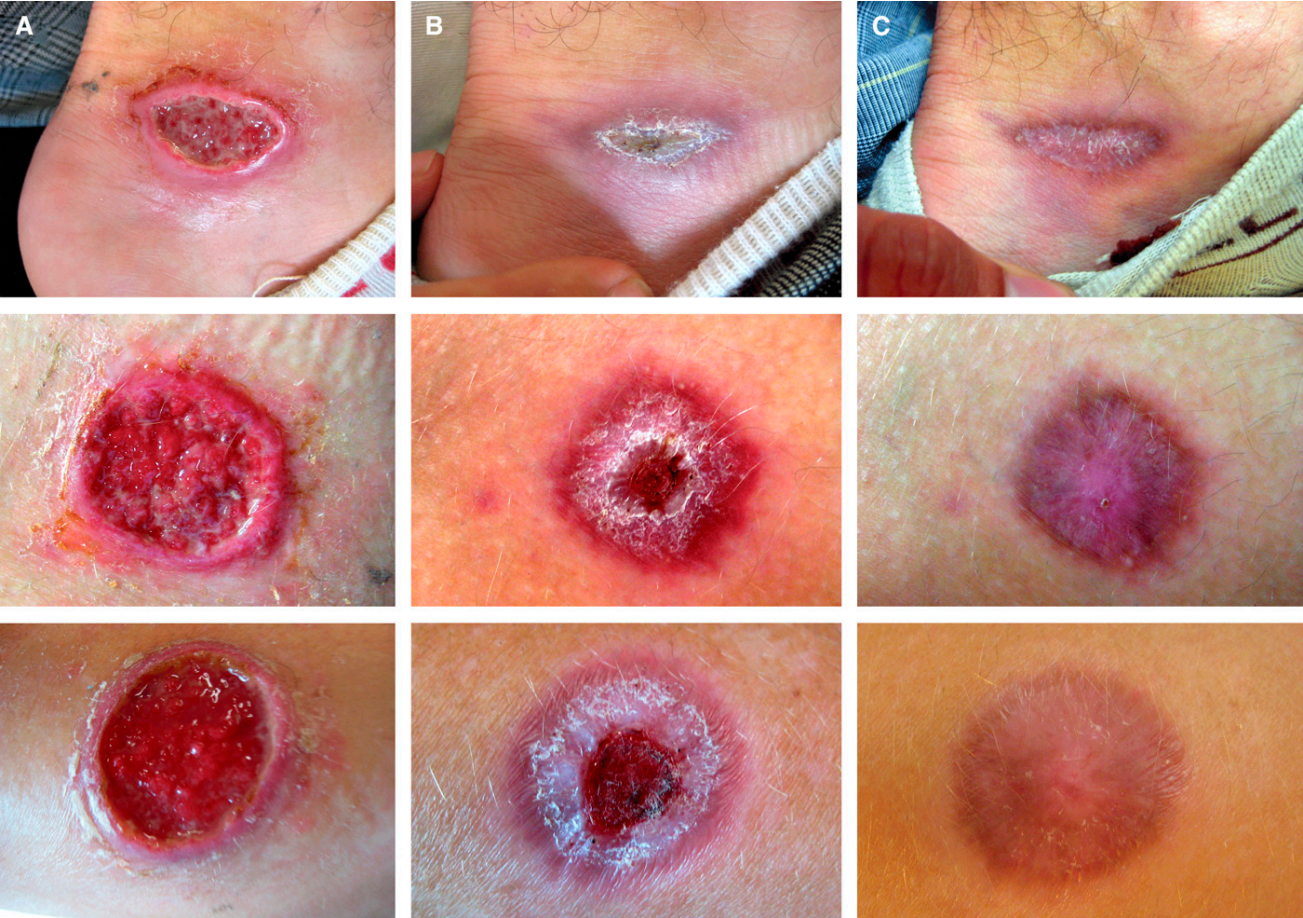
Follow-up presentation of New World cutaneous leishmaniasis. **A**, Inflammatory accentuation of the lesions' ring walls 2 weeks after start of miltefosine treatment. **B**, Wounds are nearly fully covered by scar tissue 3 weeks after cessation of miltefosine therapy. **C**, Resolution of lesions 7 weeks after the end of treatment. Upper row, lesion on the ankle; middle row, lesion on the thigh; lower row, lesion on the forearm. *Leishmania* immunoblot was positive in this patient at initial presentation. This figure appears in color at www.ajtmh.org.

An Afghan migrant who had returned from a visit to the Middle East 2 years before was seen with a non-healing painless lesion on the forearm (Figure 1A). A skin biopsy showed intracellular *Leishmania* parasites in the subcutis (Figure 1B and C), and the polymerase chain reaction (PCR) was positive for *Leishmania tropica*. At the same time, a German traveler who had returned from a vacation to Central America 1 month before was seen with progressively ulcerating painless lesions on the ankle, thigh, and forearm (Figure 2A). Scarification of the lesions' margins showed sparse *Leishmania* amastigotes (Figure 2B and C) and the PCR from biopsies was positive for *L*. *braziliensis*. A 28-day treatment with oral miltefosine (2 mg and 2.5 mg/kg, respectively) was initiated in both individuals and the patients were seen at intervals of several weeks (Figures 3 and 4). All lesions healed, but both patients developed nausea and elevated liver function tests during pharmacotherapy. The highest values of aspartate aminotransferase (AST) and alanine aminotransferase (ALT) were 66 U/L and 44 U/L in the female migrant and 73 U/L and 33 U/L in the male traveler, respectively. Bilirubin levels were normal in both patients, but the male patient developed γGT levels of 113 U/L. All values returned to normal 2 and 3 weeks after the end of treatment, respectively. Neither patient's lesions showed clinical relapse when examined 4 months after the end of therapy. Miltefosine (hexadecylphosphocholine) has been shown to be effective in cutaneous and visceral leishmaniasis.^1^ At least some strains of *L. braziliensis*, a species that can cause both cutaneous and mucocutaneous disease in the New World, have demonstrated a decreased sensitivity to the drug *in vitro*, however.^2^
